# The association of the atherogenic index of plasma with hypertension, diabetes, and their comorbidities in Chinese middle-aged and elderly people: a cross-sectional study from CHARLS

**DOI:** 10.3389/fnut.2025.1607601

**Published:** 2025-09-22

**Authors:** Yuan-Feng Zhou, Xiang-Tao Zhang, Qing-Tian Zeng, Hua-Bin He

**Affiliations:** ^1^Department of Cardiology, Jiujiang Third People’s Hospital, Jiujiang, China; ^2^Department of Cardiovascular Medicine, The Second Affiliated Hospital of Nanchang University, Nanchang, China; ^3^Department of Cardiology, Jiujiang First People’s Hospital, Jiujiang, China

**Keywords:** atherogenic index of plasma, hypertension, diabetes, comorbidities, CHARLS

## Abstract

**Background:**

The atherogenic index of plasma (AIP) is acknowledged as a contemporary indicator of insulin resistance. Previous research on AIP and metabolism-related diseases was limited and primarily concentrated on individual diseases. The aim of this investigation was to systematically examine the relationship among AIP and hypertension, diabetes, and their comorbidities.

**Methods:**

This study employed a cross-sectional design. Using data from the China Health and Retirement Longitudinal Study (CHARLS) of 2011, we conducted a systematic investigation of the association between AIP and the risk of hypertension, diabetes, and their comorbidities through restricted cubic spline plots (RCS) and multiple multivariate logistic regression. Additionally, receiver operating characteristic curves (ROC) were employed to assess AIP’s predictive validity for these conditions.

**Results:**

This study comprised 8,450 participants, with an average age of 59.57 years. The prevalence of hypertension, diabetes, and their comorbidities were 40.98, 15.62, and 8.52%, respectively. The RCS demonstrated a non-linear positive correlation between the AIP and these disorders. For each unit increased in AIP, the risk of hypertension, diabetes, and comorbidities elevated by 0.63-fold, 2.55-fold, and 2.75-fold, respectively. The ROC analysis demonstrated that AIP outperformed traditional lipid parameters in predicting both diabetes and comorbidities risk (AUC: 0.6465, 0.6725).

**Conclusion:**

This study demonstrated that heightened AIP was strongly linked to a high risk of hypertension, diabetes, and comorbidities among middle-aged and elderly Chinese individuals.

## 1 Introduction

Hypertension and diabetes are the most common chronic non-communicable diseases, and they are also important preventable risk factors for all-cause deaths and cardiovascular deaths worldwide (1, 2). Researchers find that there are common metabolic pathways between hypertension and diabetes, resulting in an interplay between them (3–5). The simultaneous presence of hypertension and diabetes presents a higher health risk compared to each condition individually (4–6). Globally, the combined burden of hypertension, diabetes, and their comorbidities poses a substantial health and economic challenge. Therefore, early screening for associated risk factors and implementing preventive measures are critical.

The atherogenic index of plasma (AIP) has been proposed as a novel marker for evaluating lipid metabolism disorders (7). However, growing evidence indicates that AIP serves as an effective indicator of insulin resistance (IR) and a significant predictor of diabetes (8, 9). For example, the study of Yin et al. (8) showed that every one-unit increment in AIP was significantly associated with a 29% elevated risk of IR (OR = 1.29, 95% CI: 1.26–1.32) and an 18% increased likelihood of diabetes (OR = 1.18, 95% CI: 1.15–1.22). Furthermore, the longitudinal study of Zhou et al. (10) revealed that higher AIP levels significantly elevated type 2 diabetes mellitus (T2DM) incidence (HR = 1.763, 95% CI 1.210–2.568). However, potentially attributable to variations in the study population, prior research yielded incongruent findings concerning the association between AIP and hypertension (11–13). Choudhary et al. (12) reported a significant association between the AIP and arterial stiffness, yet found no statistically significant correlation with blood pressure levels. In contrast, the research of Mo et al. (14) and Yuan et al. (15) revealed that elevated AIP levels were significantly linked to an increased risk of hypertension.

The IR serves as a shared pathophysiological foundation for both hypertension and diabetes. However, the potential impact of elevated AIP levels on the comorbidities of hypertension and diabetes risk remained underexplored. Therefore, the goal of this study was to systematically assess the association among AIP, hypertension, diabetes, and their comorbidities risk.

## 2 Methods

### 2.1 Study population

The data of the individuals in this study were sourced from the China Health and Retirement Longitudinal Study (CHARLS), a comprehensive national survey led by Peking University that began in 2011. The research aimed at those aged ≥45 years in 450 villages and communities throughout 28 provinces, 150 counties, and districts in China through multi-stage stratified probability proportional sampling, and conducting follow-up surveys every 2 years. Five surveys had been completed (2011, 2013, 2015, 2018, and 2020). The Peking University Biomedical Ethics Committee approved the study by the Declaration of Helsinki (No. IRB00001052-11015), and each participant executed an informed consent form.

**Figure 1:**
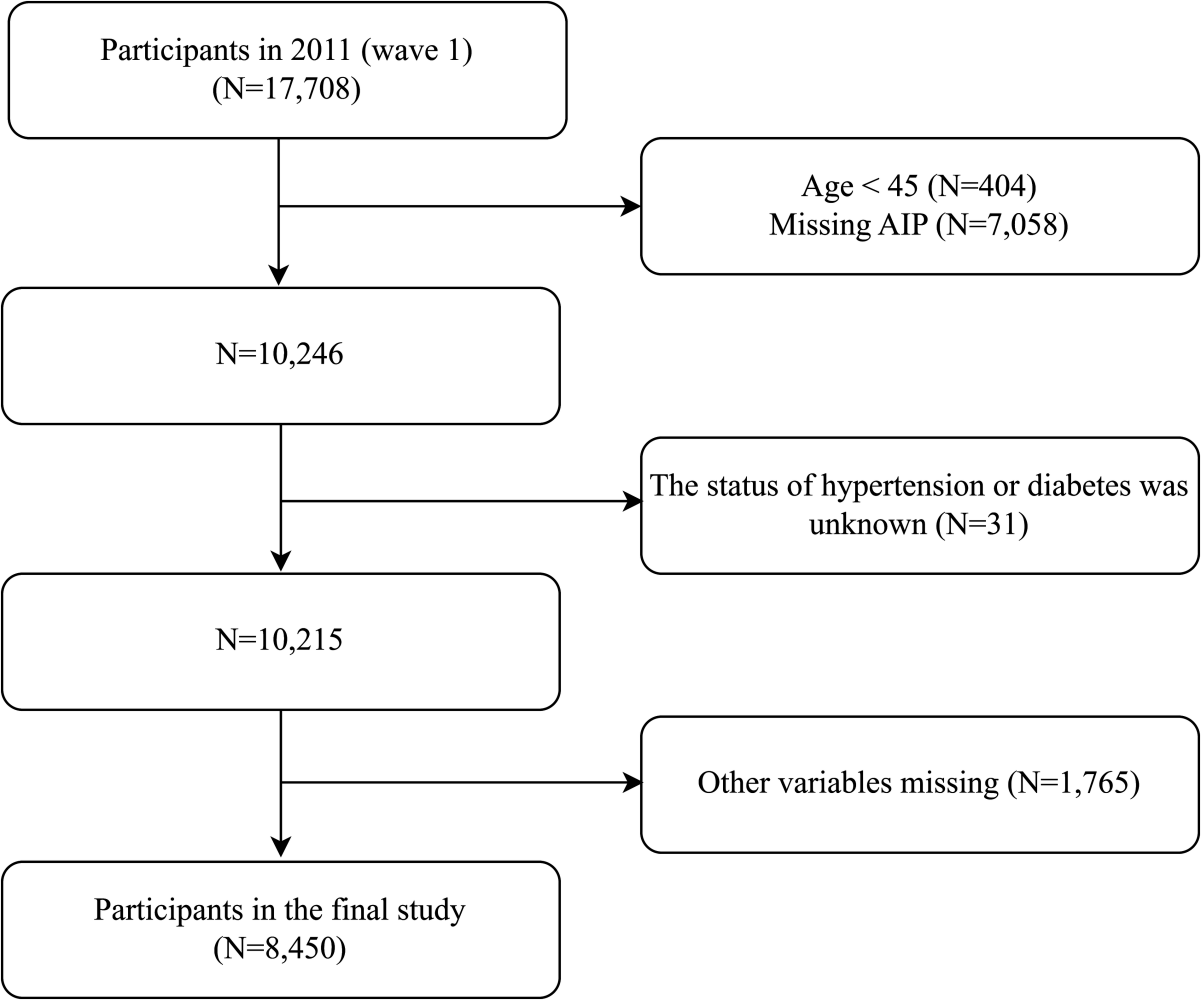
Flowchart of the study population. AIP, atherogenic index of plasma.

The data of the individuals in this study were sourced from CHARLS in 2011. The inclusion criteria were: (1) age ≥ 45 years, and (2) availability of blood test and physical examination data. The exclusion criteria were: (1) missed AIP data, (2) the status of hypertension or diabetes was unknown, and (3) missed other covariates. A total of 8,450 participants were included in the final study. Detailed information was shown in Figure 1. The reporting of this study adhered to the STROBE (Strengthening the Reporting of Observational Studies in Epidemiology) Checklist for cross-sectional studies.

### 2.2 Demographic characteristics and laboratory tests

Demographic characteristics were collected by trained professionals through questionnaires, including age, sex, education level (categorized as junior high school and below, high school and below, college and above), marital status (yes/no), place of residence (rural/urban), height (m), weight (kg), using lipid-lowing drugs (yes/no), using hypoglycemic drugs (yes/no), using antihypertensive drugs (yes/no), and smoking and drinking status (yes/no, determined based on whether the respondent was smoking or drinking at the time of the survey). BMI (body mass index) equals weight divided by height squared.

The fasting blood samples were collected by staff from the Chinese Center for Disease Control and Prevention (CDC). Complete blood count (CBC) tests were conducted using automated analyzers at county CDC stations or town/village health centers. Some blood samples were transported to the Beijing CDC within 2 weeks for further testing of other indices, including fasting blood glucose (FBG), hemoglobin A1c (HbA1c), total cholesterol (TC), triglycerides (TG), high-density lipoprotein cholesterol (HDL-C), low-density lipoprotein cholesterol (LDL-C), C-reactive protein (CRP), and serum creatinine (Scr). AIP = log (TG (mmol/L)/HDL-C (mmol/L)) (8). RC (mmol/L) = TC (mmol/L) − HDL-C (mmol/L) − LDL-C (mmol/L) (16); NHDL-C (Non-HDL-C) = TC (mmol/L) − HDL-C (mmol/L) (17).

### 2.3 Diagnosis of chronic diseases

The diagnosis of chronic diseases was made through standardized questionnaires, blood tests, and physical examinations. A diagnosis was confirmed if one of the following criteria was met. Hypertension: (1) self-reported hypertension; (2) average blood pressure ≥140/90 mmHg (18). Diabetes: (1) self-reported diabetes; (2) fasting blood ≥7 mmoL/L; (3) non-fasting blood glucose ≥11.1 mmoL/L; and (4) HbA1c ≥6.5% (19). Dyslipidemia: (1) self-reported dyslipidemia; (2) hematology tests were according to the Chinese Lipid Management Guidelines (version 2023) (20). The comorbidities were referred to as hypertension and diabetes mellitus. The estimated glomerular filtration rate (eGFR) was calculated using the modification of diet in renal disease (MDRD) equation (21); the eGFR <60 mL/min/1.73 m^2^ was taken as indicative of chronic renal insufficiency (CKI). The diagnosis of cardiovascular disease (CVD) was determined based on questionnaire responses to the following inquiries: “Has a doctor ever informed you that you have had heart disease?” and “Has a doctor ever informed you that you have had a stroke?”

### 2.4 Data analysis

Participants were categorized into four groups according to AIP. Normality of continuous variables was assessed using the Kolmogorov–Smirnov test. According to the test results, the continuous variables were expressed as mean ± standard deviation (M ± SD) or median (interquartile range), with comparisons between groups performed using the one-way analysis of variance (ANOVA) or Mann–Whitney *U* test. Categorical variables were expressed as absolute numbers (percentages), and comparisons between groups were made using the chi-square test. The correlation among AIP, hypertension, diabetes, and comorbidities was assessed by restricted cubic spline plots (RCS). Employing multiple logistic and ordered logistic regression to analyze the effect of dose in AIP and hypertension, diabetes, and their comorbidities. The selection of confounders was informed by prior research, clinical outcomes, or a variation in effect estimate exceeding 10%. Model I was unadjusted. Model II adjusted for age, sex, BMI, marital status, education, and residence. Model III expanded on Model II by additionally adjusting for smoking, drinking, dyslipidemia, lipid-lowering drugs, CRP, and CKI (additionally adjusted the antihypertensive drugs for diabetes and hypoglycemic drugs for hypertension). Results were expressed as odds ratios (OR) and 95% confidence intervals (CI). The predictive capacity of AIP and the traditional lipid metrics for hypertension, diabetes, and comorbidities was evaluated by the receiver operating characteristic curve (ROC). The stability of the connection between AIP and the three statuses across various populations was evaluated by subgroup analysis and interaction tests, utilizing subgroup characteristics including age, sex, BMI, residence, dyslipidemia, using lipid-lowering drugs, using hypoglycemic drugs, using antihypertensive drugs, CVD, CKI, smoking, and drinking status. Data was analyzed using R.studio (version 4.3.3) and EmpowerStats (version 4.0). The statistical test indicated that *p* < 0.05 on both sides was deemed statistically significant.

## 3 Results

### 3.1 The characteristics of participants

This study involved 8,450 participants with a mean age of 59.57 years. Among them, 3,463 (40.98%) had hypertension, 1,320 (15.62%) had diabetes, and 720 (8.52%) had both hypertension and diabetes. Participants were categorized into four groups according to their AIP levels as shown in Table 1. Relative to the lowest AIP group, participants in the highest group were significantly younger and included a higher proportion of females. They also exhibited elevated levels of BMI, CRP, FBG, HbA1c, TG, RC, NHDL-C, LDL-C, and TC, but lower levels of eGFR and HDL-C (all *p* < 0.001). Additionally, individuals in the highest AIP group had higher educational attainment and were more likely to reside in urban areas (all *p* < 0.001). This group also showed lower rates of smoking and drinking, yet had greater use of lipid-lowering, hypoglycemic, and antihypertensive medications (all *p* < 0.001). Furthermore, they had a significantly higher prevalence of dyslipidemia, hypertension, diabetes, and comorbidities (all *p* < 0.001).

**Table 1:** Participant characteristics grouped according to AIP.

AIP quartile	Total	AIP				*p*-value
		Q1 (−1.19, −0.24)	Q2 (−0.24, −0.04)	Q3 (−0.04, 0.19)	Q4 (0.19, 1.53)	
*N*	8,450	2,113	2,112	2,110	2,115	
Age (years)	59.57 ± 9.41	60.16 ± 9.84	59.78 ± 9.52	59.56 ± 9.28	58.78 ± 8.92	<0.001
Gender (female, *n* %)	4,533 (53.64)	1,028 (48.65)	1,122 (53.12)	1,200 (56.87)	1,183 (55.93)	<0.001
BMI (kg/m^2^)	23.52 ± 3.91	21.88 ± 3.31	22.93 ± 3.75	24.06 ± 3.83	25.22 ± 3.90	<0.001
Married (yes, *n* %)	7,378 (87.31)	1,833 (86.75)	1,823 (86.32)	1,847 (87.54)	1,875 (88.65)	0.111
Education (*n* %)						<0.001
Primary and below	5,965 (70.59)	1,545 (73.12)	1,522 (72.06)	1,470 (69.67)	1,428 (67.52)	
High school and below	2,369 (28.04)	545 (25.79)	557 (26.37)	618 (29.29)	649 (30.69)	
Above bachelor	116 (1.37)	23 (1.09)	33 (1.56)	22 (1.04)	38 (1.80)	
Residence (yes, *n* %)						<0.001
Rural	5,437 (64.34)	1,531 (72.46)	1,392 (65.91)	1,319 (62.51)	1,195 (56.50)	
Urban	3,013 (35.66)	582 (27.54)	720 (34.09)	791 (37.49)	920 (43.50)	
CRP (mg/L)	1.04 (0.55–2.18)	0.76 (0.45–1.74)	0.91 (0.52–1.91)	1.11 (0.59–2.25)	1.36 (0.74–2.78)	<0.001
FBG (mg/dL)	109.78 ± 34.82	102.76 ± 22.36	105.54 ± 27.39	109.19 ± 33.82	121.62 ± 47.40	<0.001
HbA1C (%)	5.29 ± 0.82	5.18 ± 0.63	5.22 ± 0.70	5.30 ± 0.82	5.46 ± 1.03	<0.001
eGFR (mL/min/1.73m^2^)	97.92 ± 24.10	100.83 ± 23.61	98.86 ± 23.72	96.47 ± 22.83	95.55 ± 25.81	<0.001
TC (mmol/L)	5.02 ± 0.99	4.89 ± 0.90	4.94 ± 0.97	5.02 ± 0.96	5.24 ± 1.09	<0.001
HDL-C (mmol/L)	1.33 ± 0.39	1.74 ± 0.37	1.41 ± 0.26	1.21 ± 0.22	0.96 ± 0.21	<0.001
LDL-C (mmol/L)	3.04 ± 0.91	2.89 ± 0.77	3.12 ± 0.87	3.21 ± 0.87	2.95 ± 1.05	<0.001
TG (mmol/L)	1.18 (0.84–1.71)	0.69 (0.59–0.81)	1.00 (0.88–1.15)	1.39 (1.21–1.59)	2.29 (1.87–3.04)	<0.001
NHDL-C (mmol/L)	3.69 ± 0.99	3.14 ± 0.79	3.53 ± 0.88	3.82 ± 0.88	4.28 ± 1.04	<0.001
RC (mmol/L)	0.50 (0.29–0.81)	0.23 (0.13–0.36)	0.38 (0.27–0.53)	0.58 (0.45–0.74)	1.09 (0.82–1.52)	<0.001
Smoking (yes, *n* %)	2,537 (30.02)	696 (32.94)	674 (31.91)	585 (27.73)	582 (27.52)	<0.001
Drinking (yes, *n* %)	2,781 (32.91)	858 (40.61)	672 (31.82)	606 (28.72)	645 (30.50)	<0.001
Dyslipidemia (yes, *n* %)	3,398 (40.21)	291 (13.77)	492 (23.30)	806 (38.20)	1,809 (85.53)	<0.001
Using lipid-lowering drugs (yes, *n* %)	474 (5.61)	53 (2.51)	82 (3.88)	125 (5.92)	214 (10.12)	<0.001
Diabetes (yes, *n* %)	1,320 (15.62)	193 (9.13)	242 (11.46)	329 (15.59)	556 (26.29)	<0.001
Using hypoglycemic drugs (yes, *n* %)	320 (3.79)	50 (2.37)	52 (2.46)	85 (4.03)	133 (6.29)	<0.001
Hypertension (yes, *n* %)	3,463 (40.98)	672 (31.80)	783 (37.07)	939 (44.50)	1,069 (50.54)	<0.001
Using antihypertensive drugs (yes, *n* %)	1,705 (20.18)	251 (11.88)	354 (16.76)	489 (23.18)	611 (28.89)	<0.001
CVD (yes, *n* %)	1,139 (13.48)	199 (9.42)	257 (12.17)	318 (15.07)	365 (17.26)	<0.001
Comorbidities (yes, *n* %)	720 (8.52)	90 (4.26)	112 (5.30)	179 (8.48)	339 (16.03)	<0.001

AIP, atherogenic index of plasma; BMI, body mass index; CRP, c-reactive protein; CKD, chronic kidney disease; CVD, cardiovascular disease; eGFR, estimated glomerular filtration rate; FPG, fasting plasma glucose; HDL-C, high-density lipoprotein cholesterol; HbA1c, hemoglobin A1c; LDL-C, low-density lipoprotein cholesterol; NHDL-C, non-high-density lipoprotein cholesterol; RC, residual cholesterol; TC, total cholesterol; Scr, serum creatinine; TG, triglyceride.

### 3.2 Correlation between AIP and hypertension, diabetes, and their comorbidities

**Figure 2:**
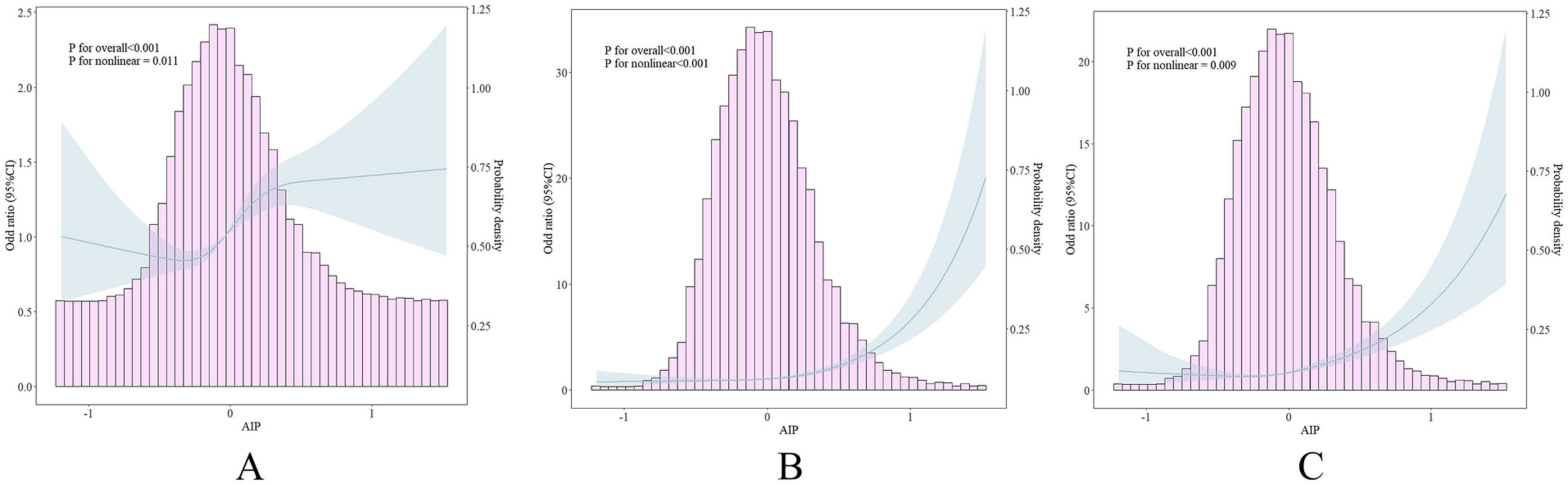
The association between AIP, hypertension, diabetes, and their comorbidities. Adjusted for age, gender, BMI, marital status, education, residence, smoking, drinking, dyslipidemia, using lipid-lowering drugs, CRP, and CKI (additionally adjusted the antihypertensive drugs for diabetes and hypoglycemic drugs for hypertension). **(A)** For hypertension; **(B)** for diabetes; **(C)** for comorbidities. AIP, atherogenic index of plasma; BMI, body mass index; CKI, chronic renal insufficiency; CPR, C-reactive protein.

The RCS was employed to investigate the relationship among the AIP and hypertension, diabetes, and comorbidities. As illustrated in Figure 2, there was a positive connection between AIP, hypertension (Figure 2A), diabetes (Figure 2B), and their comorbidities (Figure 2C) (all *p* for overall <0.05). And the relationships were nonlinear (*p* for nonlinear were 0.011, <0.001, and 0.009). Subsequently, we constructed several regression models to evaluate the dose–response relationship among AIP and hypertension, diabetes, and their comorbidities, illustrated in Table 2. In the fully adjusted model (Model III), elevated levels of the AIP were independently associated with an increased risk of hypertension, diabetes, and their comorbidities. Specifically, for a one-unit increase of AIP, the risks of hypertension, diabetes, and comorbidities increased by 0.63-fold (OR: 1.63, 95% CI: 1.37, 1.95), 2.55-fold (OR: 3.55, 95% CI: 2.84, 4.44), and 2.75-fold (OR: 3.75, 95% CI: 2.82, 4.99), respectively. In comparison to the first quartile, the risk of hypertension escalated by 0.51-fold (OR: 1.51, 95% CI:1.29, 1.77), diabetes by 1.14-fold (OR: 2.14, 95% CI:1.73, 2.65), and comorbidities by 1.31-fold (OR: 2.31, 95% CI: 1.73, 3.08) in the fourth quartile.

**Table 2:** Association of AIP and hypertension, diabetes, and their comorbidities.

Exposure	Adjusted Model I OR (95%CI)	*p*-value	Adjusted Model II OR (95%CI)	*p*-value	Adjust Model III OR (95%CI)	*p*-value
Hypertension						
AIP	2.48 (2.16, 2.84)	<0.0001	1.79 (1.54, 2.08)	<0.0001	1.63 (1.37, 1.95)	<0.0001
AIP quartile						
Q1	Ref.		Ref.		Ref.	
Q2	1.26 (1.11, 1.43)	0.0003	1.14 (0.99, 1.30)	0.0653	1.13 (0.98, 1.29)	0.0863
Q3	1.72 (1.52, 1.95)	<0.0001	1.39 (1.22, 1.59)	<0.0001	1.35 (1.18, 1.56)	<0.0001
Q4	2.19 (1.93, 2.48)	<0.0001	1.63 (1.42, 1.87)	<0.0001	1.51 (1.29, 1.77)	<0.0001
*p* for trend		<0.0001		<0.0001		<0.0001
Diabetes						
AIP	5.49 (4.59, 6.56)	<0.0001	4.76 (3.95, 5.75)	<0.0001	3.55 (2.84, 4.44)	<0.0001
AIP quartile						
Q1	Ref.		Ref.		Ref.	
Q2	1.29 (1.05, 1.57)	0.0131	1.21 (0.99, 1.48)	0.0662	1.16 (0.94, 1.42)	0.1611
Q3	1.84 (1.52, 2.22)	<0.0001	1.62 (1.33, 1.96)	<0.0001	1.41 (1.16, 1.73)	0.0007
Q4	3.55 (2.97, 4.23)	<0.0001	2.97 (2.47, 3.57)	<0.0001	2.14 (1.73, 2.65)	<0.0001
*p* for trend		<0.0001		<0.0001		<0.0001
Comorbidities						
AIP	6.32 (5.08, 7.88)	<0.0001	5.13 (4.05, 6.50)	<0.0001	3.75 (2.82, 4.99)	<0.0001
AIP quartile						
Q1	Ref.		Ref.		Ref.	
Q2	1.26 (0.95, 1.67)	0.1125	1.11 (0.83, 1.48)	0.4799	1.07 (0.80, 1.43)	0.6454
Q3	2.08 (1.61, 2.70)	<0.0001	1.68 (1.29, 2.20)	<0.0001	1.46 (1.10, 1.92)	0.0079
Q4	4.29 (3.37, 5.46)	<0.0001	3.18 (2.47, 4.10)	<0.0001	2.31 (1.73, 3.08)	<0.0001
*p* for trend		<0.0001		<0.0001		<0.0001

Adjusted Model I: none adjusted. Adjusted Model II: adjusted for age, gender, BMI, marital status, education, and residence. Adjust Model III: adjusted for age, gender, BMI, marital status, education, residence, smoking, drinking, dyslipidemia, using lipid-lowering drugs, CRP, and CKI (additionally adjusted the antihypertensive drugs for diabetes and hypoglycemic drugs for hypertension). AIP, atherogenic index of plasma; BMI, body mass index; CKI, chronic kidney insufficiency; CRP, C-reactive protein.

**Figure 3:**
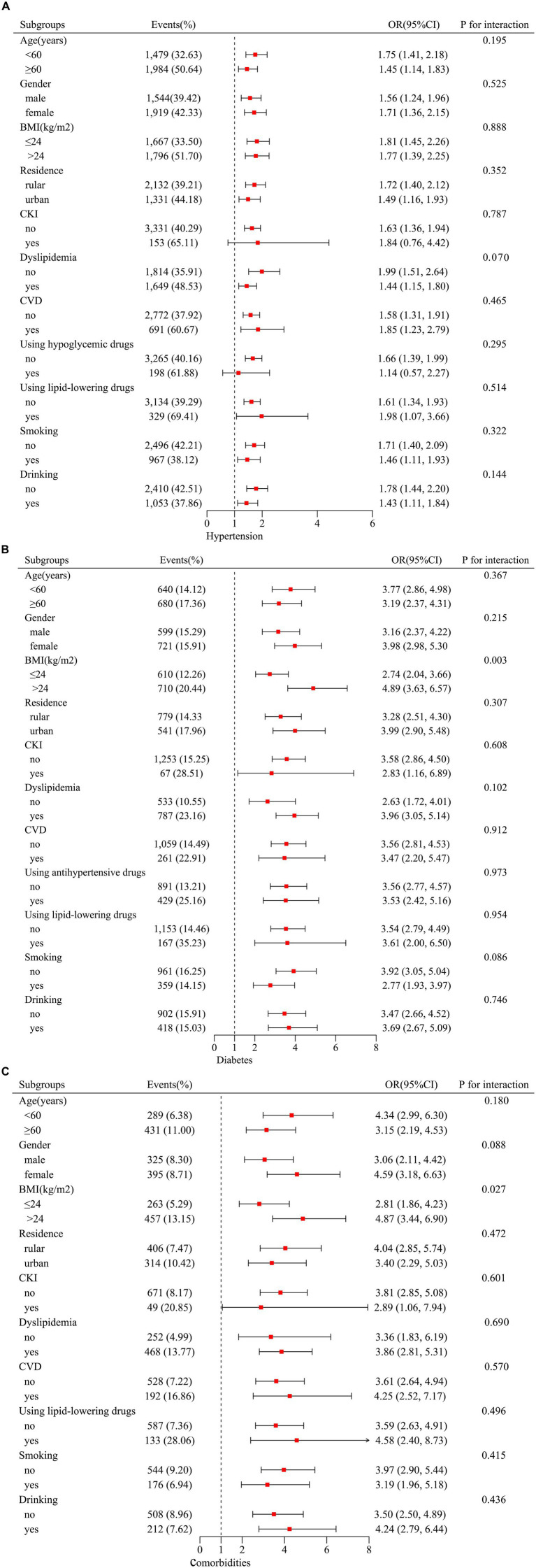
Subgroup analyses between AIP, hypertension, diabetes, and their comorbidities. Stratification variables included age, sex, BMI, residence, dyslipidemia, using lipid-lowering drugs, using hypoglycemic drugs, using antihypertensive drugs, CVD, CKI, smoking, and drinking status. Adjusted for age, gender, BMI, marital status, education, residence, smoking, drinking, dyslipidemia, using lipid-lowering drugs, CRP, and CKI (additionally adjusted the antihypertensive drugs for diabetes and hypoglycemic drugs for hypertension). **(A)** For hypertension; **(B)** for diabetes; **(C)** for comorbidities. Stratification variables were not adjusted. AIP, atherogenic index of plasma; BMI, body mass index; CKI, chronic renal insufficiency; CPR, C-reactive protein.

To assess the robustness of our findings, the subgroup analyses and interaction tests were conducted. The grouping variables include age, sex, BMI, residence, dyslipidemia, use of lipid-lowering, hypoglycemic, antihypertensive drugs, CVD, CKI, and smoking and drinking states. As shown in Figure 3, the relationship among AIP, hypertension, and the comorbidities remained consistent across all subgroups (*p* for interaction >0.05). However, the association between AIP and diabetes, the comorbidities were notably stronger in individuals with a BMI >24 kg/m^2^ (*p* for interaction <0.05).

### 3.3 Predictive ability of AIP on hypertension, diabetes, and comorbidities

**Figure 4:**
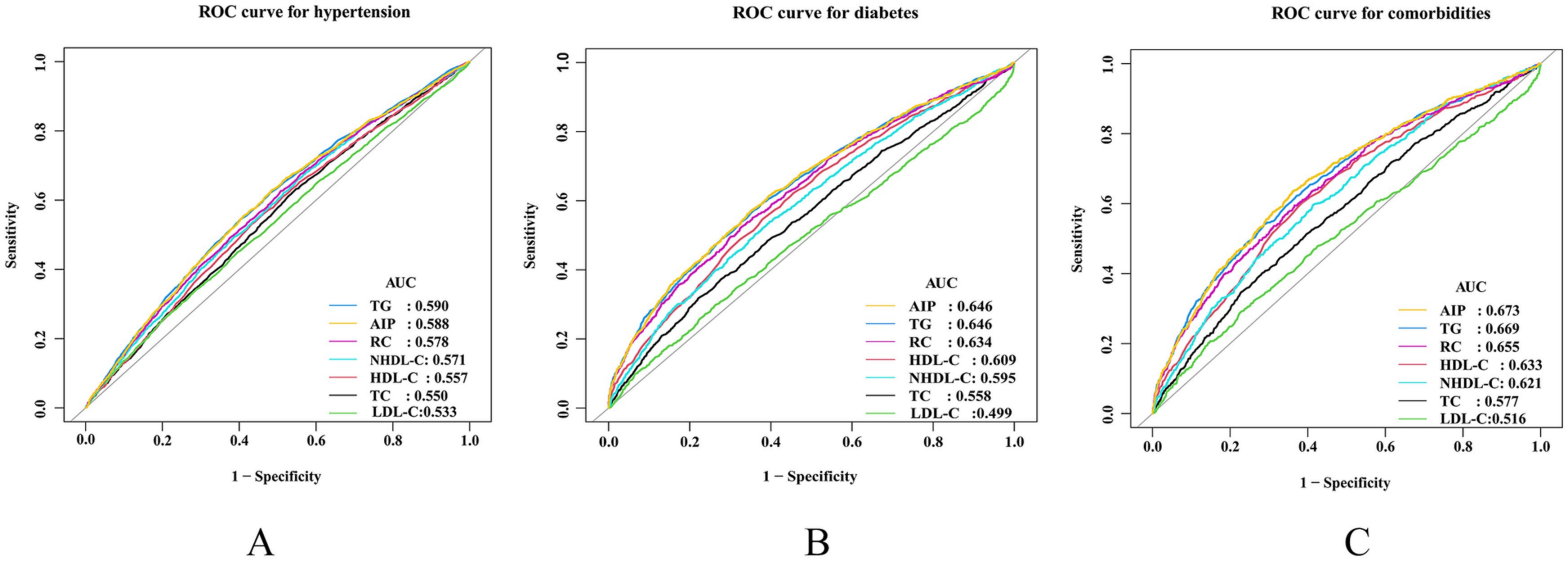
Evaluate the predictive ability of AIP, traditional lipid metrics for hypertension, diabetes, and their comorbidities. **(A)** For hypertension; **(B)** for diabetes; **(C)** for comorbidities. AIP, atherogenic index of plasma; AUC, area under the curve; HDL-C, high-density lipoprotein cholesterol; LDL-C, low-density lipoprotein cholesterol; NHDL-C, non-high-density lipoprotein cholesterol; RC, residual cholesterol; TC, total cholesterol; TG, triglyceride.

We employed ROC to assess the prediction capacity of AIP and traditional lipid metrics for hypertension, diabetes, and comorbidities. As shown in Figure 4, AIP demonstrated good predictive ability for diabetes (Figure 4B) and comorbidities (Figure 4C), with area under the curve (AUC) values of 0.6465 and 0.6725, respectively. The predictive ability of AIP for hypertension was lower than TG (0.5882 vs. 0.5902) (Figure 4A). The optimal predictive values of AIP were −0.074, 0.0540, and 0.0542, respectively (Table 3).

**Table 3:** The predictive ability of AIP, traditional lipid metrics for hypertension, diabetes, and their comorbidities.

Test	AUC	95% CI low	95% CI up	Best threshold	Specificity	Sensitivity
Hypertension						
TG	0.5902	0.5779	0.6025	1.2340	0.5968	0.5446
AIP	0.5882	0.5759	0.6005	−0.0740	0.5179	0.6260
RC	0.5781	0.5657	0.5904	0.4499	0.4919	0.6263
NHDL	0.5714	0.5591	0.5838	3.3891	0.4484	0.6587
HDL-C	0.5570	0.5446	0.5694	1.2547	0.5687	0.5302
TC	0.5498	0.5374	0.5622	4.7538	0.4524	0.6321
LDL-C	0.5333	0.5207	0.5458	3.6441	0.7945	0.2599
Diabetes						
AIP	0.6465	0.6296	0.6634	0.0540	0.6498	0.5705
TG	0.6455	0.6287	0.6624	1.3639	0.6407	0.5735
RC	0.6338	0.6168	0.6508	0.6498	0.6732	0.5235
HDL-C	0.6094	0.5925	0.6263	1.1947	0.6216	0.5455
NHDL	0.5948	0.5778	0.6118	3.7990	0.6035	0.5394
TC	0.5584	0.5410	0.5757	5.6435	0.7797	0.3167
LDL-C	0.4988	0.4808	0.5168	3.5141	0.7356	0.2992
Comorbidities						
AIP	0.6725	0.6511	0.6939	0.0542	0.6392	0.6347
TG	0.6685	0.6470	0.6901	1.5438	0.7106	0.5417
RC	0.6551	0.6335	0.6768	0.6198	0.6347	0.5944
HDL-C	0.6326	0.6111	0.6541	1.1947	0.6140	0.6028
NHDL	0.6214	0.6000	0.6427	3.7690	0.5855	0.5972
TC	0.5767	0.5545	0.5989	5.4636	0.7173	0.4014
LDL-C	0.5161	0.4923	0.5398	3.6241	0.7709	0.2917

AIP, atherogenic index of plasma; AUC, area under the curve; HDL-C, high-density lipoprotein cholesterol; LDL-C, low-density lipoprotein cholesterol; NHDL-C, non-high-density lipoprotein cholesterol; RC, residual cholesterol; TC, total cholesterol; TG, triglyceride; 95% CI, 95% confidence interval.

## 4 Discussion

Assessing risk factors for hypertension and diabetes is vital for early intervention, improving health outcomes, and decreasing the likelihood of mortality. The present study revealed a significant positive association among AIP, hypertension, diabetes, and their comorbidities, with these findings remaining stable even after adjusting for potential factors. The results of subgroup analysis and interaction test revealed that the association between AIP and hypertension and the comorbidities was consistent across most subgroups. However, the association between AIP and diabetes, comorbidities was more pronounced in individuals with BMI >24 kg/m^2^.

IR is widely recognized as a key pathophysiological mechanism underlying the development of both hypertension and diabetes, with dysregulated lipid metabolism serving as a prominent metabolic feature (22). Prior studies revealed a higher prevalence of lipid abnormalities among hypertension and diabetes (23, 24). For instance, Wyszyńska et al. (25) reported marked elevations in TC, LDL-C, and TG levels in hypertensive individuals. Similarly, Choudhury et al. (26) observed approximately 1.1-fold increases in TC and TG, a 1.2-fold increase in LDL-C, and a 1.1-fold reduction in HDL-C compared to normotensive counterparts. Elevated TG levels lead to an excessive accumulation of free fatty acids (FFA), which impair insulin sensitivity and induce lipotoxicity, thereby disrupting cellular function and pancreatic β-cell integrity, promoting apoptosis, and accelerating IR progression (15, 27, 28). At the same time, LDL-C penetrates the subendothelial space and undergoes oxidative modification, resulting in the formation of oxidized LDL (ox-LDL), which will activate vascular smooth muscle cell proliferation and promote extracellular matrix deposition, both of which are pivotal events in the development of atherosclerosis and the progressive narrowing of the vascular lumen (29). Besides, the reduction in HDL-C diminishes its vasoprotective capacity and impairs glucose uptake in skeletal muscle, further worsening IR (30). Additionally, RC enters the vascular wall via scavenger receptors, promoting foam cell formation and exacerbating vascular injury and atherosclerosis (31, 32).

The AIP is derived by calculating the logarithm of the ratio between TG and HDL-C, reflecting the dynamic interplay between atherogenic and protective lipid components. Besides, the AIP is regarded as a surrogate marker for estimating plasma concentrations of small dense low-density lipoprotein (sdLDL) particles, which exhibit greater atherogenic potential compared to other LDL subfractions (7, 33). Elevated AIP is strongly associated with endothelial dysfunction, characterized by diminished nitric oxide (NO) synthesis, release, and bioavailability. This impairment compromises the regulation of vascular tone, particularly the balance between vasodilation and vasoconstriction, thereby contributing to the initiation and progression of hypertension (34, 35). However, multiple studies had examined the relationship between AIP and hypertension, producing disparate findings. The study of Onat et al. (36) revealed that AIP independently predicted hypertension, with markedly superior sensitivity in men relative to women. Besides, a study conducted on Japanese with normal glucose metabolism found a positive correlation between AIP and elevated blood pressure (13). In contrast, Cheng et al. (24) discovered no correlation between AIP and hypertension. The current study was based on existing research and found a significant positive link between AIP and hypertension. And the association remained stable across different subgroups.

Besides, we found that there was a positive association between AIP and diabetes, consistent with previous research. As for the extra effect of AIP on glucose metabolism, the research conducted by Zou et al. (37) proved that increased AIP affected the prognosis of individuals with prediabetes by raising their risk of developing diabetes and hindering their return to normal glucose metabolism. Furthermore, the results of a meta-analysis endorsed the utilization of AIP as a crucial metric for evaluating diabetes risk (38). Besides, the current analysis revealed a more prominent association between higher AIP levels and diabetes in the obese groups, which might be attributed to obesity and abnormal lipid metabolism exacerbating IR, thereby triggering further lipid metabolism abnormalities and creating a vicious cycle that worsens glucose metabolism disorders (39–41). The results of ROC analysis revealed that among various lipid indices, the AIP exhibited superior predictive performance for diabetes (AUC = 0.6465). Consistent with this, Tao et al. (30) and Yang et al. (42) identified the TG/HDL-C ratio as the most strongly associated non-traditional lipid parameter with both prediabetes and T2DM. Wang et al. (43) also corroborated the diagnostic utility of the TG/HDL-C ratio for diabetes, recognizing it as the most discriminative lipid parameter evaluated, with an AUC of 0.684, similar to our results.

Additionally, we also found a positive association between the AIP and the coexistence of hypertension and diabetes, especially in obese individuals, which might be related to stronger IR in obese individuals. Although ROC curve analysis indicated that AIP has appropriate power for hypertension, diabetes, and their coexistence, AIP demonstrated superior diagnostic accuracy compared to conventional lipid markers.

This study revealed a significant positive association between the AIP and the risk of both hypertension and diabetes. Nevertheless, studies investigating the impact of elevated AIP levels on blood pressure regulation and glycemic control in individuals with hypertension or diabetes remained limited. Prior investigations had reported differences in AIP levels across patient populations with varying treatment responses. For instance, Mahmood et al. (44) found elevated AIP levels in patients undergoing antihypertensive treatment relative to those who were untreated; however, the difference was not statistically significant, potentially due to limited sample size. In contrast, Rabizadeh et al. (45) found a significant increase in AIP levels among patients with poorly controlled blood pressure compared to those with effective control. Similarly, Ma et al. (46) analyzed individuals with hypertension and observed a trend of increasing AIP levels corresponding to increasing blood pressure levels. Furthermore, AIP levels tend to increase with the severity of disruptions in glucose metabolism (47). The study of Susanti et al. (48) showed that patients exhibiting poor glycemic control demonstrated significantly elevated AIP levels compared to those with well-regulated blood glucose. In the future, additional research was warranted to elucidate the influence of elevated AIP levels on blood pressure regulation and glycemic control, particularly among individuals with hypertension or diabetes.

Although lipid-lowering agents were not primarily prescribed for the direct management of hypertension or diabetes, these therapies might exert modest antihypertensive effects alongside their lipid-modifying actions. Existing evidence suggested that treatment with atorvastatin might enhance arterial compliance and significantly modulate lipid profiles by reducing TC, TG, and LDL-C, while increasing HDL-C and indirectly contributing to a reduction in AIP (49, 50). Moreover, insulin sensitizers had shown efficacy in improving IR, resulting in significant reductions in AIP levels and potentially lowering cardiovascular risk. For example, Tan et al. (51) found that pioglitazone could significantly reduce AIP levels in patients with diabetes. In addition, an interventional study conducted in China demonstrated that treatment with glucagon-like peptide-1 receptor agonists (GLP-1RAs) led to significant reductions of TG, TC, and AIP levels (52). These findings suggested that antidiabetic agents might offer therapeutic benefits in managing dyslipidemia associated with diabetes. However, in our present study, subgroup analyses and interaction tests did not reveal significant differences in the risk of hypertension, diabetes, or their comorbidities between participants receiving lipid-lowering or hypoglycemic therapies and those who did not (*p* for interaction >0.05). This might be because this study employed a cross-sectional design, with information on medication use obtained through self-administered questionnaires, which might introduce recall bias. Furthermore, the absence of detailed records on medication duration and dosage limited the assessment of treatment adherence. Consequently, to more rigorously evaluate the effects of pharmacological interventions on study outcomes, future research should prioritize prospective randomized controlled trials to generate more robust and reliable evidence.

The current study validated the effects of AIP on hypertension and diabetes in a middle-aged and elderly Chinese population and explored the association between AIP and hypertension-diabetes comorbidity for the first time. Nevertheless, the study possessed certain shortcomings. First, as a cross-sectional study, this research could not establish a causal relationship between AIP and the risk of hypertension, diabetes, or their comorbidities. Future prospective studies were needed to further investigate this association and explore whether reducing AIP levels might contribute to lowering the incidence of these conditions. Additionally, the subjects of this study were middle-aged and elderly people, and the generalizability of the results to different populations needs to be further explored. Third, the diagnosis of certain diseases and the use of medicine relied on self-reported questionnaire data, which might introduce recall bias. And the potential risk-reducing effects of lipid-lowering, antihypertensive, and antidiabetic therapies on the development of hypertension, diabetes, and their related comorbidities remained inconclusive; further research was needed. Besides, some samples were excluded due to missing data, which may have impacted the study’s results. Fifth, the predictive capability of AIP for the three metabolic abnormalities remained limited. Future research should focus on identifying more precise and easily applicable biomarkers to enhance the efficiency and clinical feasibility of disease assessment and management. Finally, the potential impact of elevated AIP levels on blood pressure and glycemic control among hypertension and diabetes remained unknown. Future in-depth research and longitudinal observation were warranted to clarify this association.

## 5 Conclusion

This study showed that heightened AIP was substantially associated with an augmented risk of hypertension, diabetes, and comorbidities. Therefore, reducing AIP levels might diminish the risk of these disorders. Despite some limitations, this research offers crucial evidence for the future management of hypertension and diabetes.

## Data Availability

The original contributions presented in the study are included in the article/supplementary material, further inquiries can be directed to the corresponding author.
